# Antibiotic Stewardship Based on Colonization with Multi-Drug-Resistant Bacteria in Liver Transplantation: A Narrative Review

**DOI:** 10.3390/microorganisms12122493

**Published:** 2024-12-03

**Authors:** Valentina Zuccaro, Paola Giordani, Francesca Binda, Erika Asperges, Elisa Farina, Mauro Viganò, Elena Gervasi, Elisabetta Pagani, Stefano Fagiuoli, Raffaele Bruno

**Affiliations:** 1 Department of Diagnostic, Paediatric, Clinical and Surgical Science, University of Pavia, 27100 Pavia, Italy; 2 Department of Infectious Diseases, Fondazione IRCCS Policlinico San Matteo, 27100 Pavia, Italy; paola.giordani01@universitadipavia.it (P.G.); e.asperges@smatteo.pv.it (E.A.); e.pagani@smatteo.pv.it (E.P.); 3 Infectious Diseases Unit, ASST Papa Giovanni XXIII, 24127 Bergamo, Italy; fbinda@asst-pg23.it (F.B.); egervasi@asst-pg23.it (E.G.); 4 Gastroenterology, Hepatology and Liver Transplantation Unit, ASST Papa Giovanni XXIII, 24127 Bergamo, Italy; efarina@asst-pg23.it (E.F.); mvigano@asst-pg23.it (M.V.); sfagiuoli@asst-pg23.it (S.F.); 5 Gastroenterology, Department of Medicine & Surgery, University Milan Bicocca, 20126 Milan, Italy

**Keywords:** liver transplantation, bacterial infections, multi-drug-resistant organism, antibiotic stewardship, colonization

## Abstract

In solid organs post-transplant, bacterial infections can complicate the course of recovery with devastating consequences, such as graft loss and death. We provide an expert review on early post-liver transplant bacterial infections, with a focus on infections with multi-drug-resistant organism (MDRO) etiologies. Best practice recommendations are derived from a combination of available evidence and expert consensus. The main challenge in managing antibiotic therapy arises in patients with severe clinical conditions but negative MDRO screening results, as well as in those with positive MDRO screening results but uncomplicated infections. With the aim of shedding light on these “gray areas”, we propose an algorithm where the patient is stratified as being at low risk or high risk of developing an MDRO infection.

## 1. Introduction

Patients with severe liver disease often suffer from infections. They can present both before and after liver transplantation, with different characteristics, and they have important consequences for both morbidity and mortality [1,2]. In this context, infections caused by multi-drug-resistant organisms (MDROs) are obviously relevant, as they usually involve fragile patients with multiple comorbidities, immune suppression, or the need for intensive care [3,4].

According to the last guidelines from the European Association for the Study of the Liver (EASL), screening for latent infections is recommended for every liver transplant (LT) candidate. Among these screening procedures, most of which are standard (e.g., HIV antibodies, Varicella Zoster Virus serologies), the only one that explicitly pertains to MDROs is the nasal/axillary swab for *Staphylococcus aureus*. There is, however, a recommendation to take into account local epidemiology when considering whether to perform extra, non-standard screenings [5]. Moreover, MDROs can be detected in the course of microbiological testing during pre-transplant infectious episodes, such as spontaneous bacterial peritonitis or sepsis.

In the post-transplant phase, bacterial infections can complicate the course of recovery with devastating consequences, such as graft loss and death. Classically, early infections are defined as those that occur in the first 30 days post-transplant and are related to surgery and hospitalization or are donor-derived [2,6]. The most common causes are bacteria from the family *Enterobacteriaceae*, and the most common syndromes include intra-abdominal, surgical site, and bloodstream infections [7,8]. There are no specific recommendations for the management of these complications; therefore, physicians must rely on clinical guidelines relating to infectious disease without a specific focus on transplanted patients.

Working as a divide, surgical prophylaxis should help to prevent post-transplant infection, taking into account screening results and pre-transplant infections. However, no specific guidance exists, and each center is left to decide on their own, hopefully considering the local epidemiology.

With all this in mind, this review aims to establish the kind of infection that afflicts liver transplant patients before and after surgery, the role of MDROs in this context, and the measures currently adopted around the world to prevent these infections. A secondary aim is to provide guidance, in the form of expert opinion, on the application of the principles of antimicrobial stewardship in the context of liver transplantation, with the final objective of improving outcomes, limiting MDRO spread, and helping to choose the most appropriate therapy.

## 2. Materials and Methods

We conducted a review of the literature using the PubMed database and searched for articles regarding liver transplantation infections and antibiotic therapy management, mainly focusing on colonization with multi-drug-resistant bacteria. The search terms used were combinations of the terms “Drug Resistance, Multiple, Bacterial” [Mesh Terms], “Liver Transplantation” [Mesh Terms], “Antibiotic Prophylaxis” [Mesh Terms] “Carrier State” [MeSH Terms], “MDRO infection”, “rectal colonization”, and “early infection”. The target population included adult patients; the pediatric population was excluded. The search period was from January 2020 to May 2024.

There were no limitations in terms of article type, except in terms of language (English only). The references of retrieved full texts were also screened to identify further studies suitable for inclusion.

## 3. Multi-Drug-Resistant Infections in Liver Transplant Recipients—Epidemiology

The incidence of LT infections, including both community-acquired and nosocomial infections, ranges widely from 33% to 68% [9]. LT infections are classified into early infections (occurring within the first month) and late infections. In the early phase, the most prevalent infections are abdominal infections; postoperative surgical site infections (SSIs); and bloodstream, urinary, and respiratory tract infections.

Bacterial abdominal infections are the most common ones following LT, accounting for 27–47% of all cases. These infections include peritonitis, superinfections with ascites, cholangitis, and hepatic abscesses. Diagnosis is typically confirmed by a positive culture of abdominal drainage fluid, identifying both Gram-positive organisms, such as *Enterococcus* and *S. aureus* (responsible for 38% and 12% of early intra-abdominal infection cases, respectively), and Gram-negative bacilli, primarily *Enterobacterales* (28%) [10]. These infections are associated with significant mortality rates.

Surgical wound infections occur in 10–37% of cases and are a major cause of prolonged hospitalization and mortality within the first year [11]. Diagnosis is primarily clinical. These issues are characterized by the appearance of local signs of inflammation or purulent drainage accompanied by systemic symptoms. Gram-positive bacteria dominate surgical site isolates, comprising 65% of cases, with coagulase-negative Staphylococci making up 23–25% and *Enterococcus* 5–25% of these isolates. Gram-negative bacilli are found in 29–48% of samples, and are mainly represented by *Enterobacteriaceae* strains (60%); polymicrobial infections are not uncommon (17% of episodes).

Bloodstream infections are common in the first month following liver transplantation. The most frequent sources of these infections include the gastrointestinal tract, urinary tract, and lower respiratory tract, and there are also infections caused by the presence of a contaminated indwelling vascular catheter (CLABSI). The likely underlying pathophysiological mechanism involves increased intestinal permeability, which leads to enhanced bacterial translocation [10]. In a study by Bucheli et al., 20% of liver transplant recipients developed enterococcal infections within the first 6 months [12]. This finding was corroborated by a study from Kim et al., which identified enterococcal bloodstream infections (BSIs) as the most common cause of BSIs [13]. However, bloodstream infections can also be caused by *S. aureus*, *Streptococcus viridans*, *Gram-negative bacilli*, and even by a polymicrobial infection. Specifically, MRSA is responsible for up to 50% of BSIs. Meanwhile, Gram-negative bacteria are an increasing source of BSIs, with rising resistance levels: resistant *E. coli* strains are found in nearly 13% of cases in some centers. Additionally, multi-drug-resistant strains have been identified in 62.5% of *A. baumannii*, 54.2% of *Stenotrophomonas maltophilia*, and 51.5% of *Pseudomonas* isolates.

Regarding catheter-related urinary tract infections, the risk significantly increases when the catheter remains in place for more than 3 days [14].

As a whole, Gram-negative bacilli have emerged as the predominant cause of infection [15]. Furthermore, there has been a notable increase in MDRO infections among cirrhotic and liver transplant patients. This rise is attributed to the widespread use of antibiotic prophylaxis, frequent hospitalizations, and higher rates of ICU admission [10]. Multiple studies have shown that infection by MDR pathogens is the primary factor affecting the early survival rate of solid organ transplantation recipients [16].

## 4. Risk Factors for Acquisition of Multi-Drug-Resistant Organisms Among Liver Transplant Recipients

Recent research has focused on identifying risk factors in post-transplant bacterial infections with the goal of addressing modifiable risks and improving transplant outcomes. In the specific field of LT, numerous risk factors for infections have been reported; however, the limited number of studies and the lack of high-level evidence make it challenging to draw definitive conclusions. Moreover, these studies mainly focus on generic risk factors, and not on MDROs.

It is worth noting that the spread of certain bacteria poses a significant threat, as many of these species carry antibiotic resistance genes (ARGs) which can be inherited vertically and also spread horizontally between bacteria by horizontal gene transfer (HGT), facilitated by mobile genetic elements (MGEs) such as plasmids, transposons, and integrons. The most common mechanisms of resistance include enzymatic degradation (e.g., carbapenemases, such as blaKPC, blaNDM, and blaOXA-48), efflux pumps that expel antibiotics from bacterial cells, target site modification (e.g., altered PBPs or ribosomal RNA), and a reduction in permeability due to porin channel mutations [17]. With this in mind, infection control measures, such as the implementation of isolation protocols to prevent the transmission of pathogenic microorganisms from colonized or infected individuals to others, currently remain the most robust strategies for the prevention of pre-transplant infection.

Risk factors can be categorized into three groups: pre-transplant, intraoperative, and post-transplant factors [18]. We summarize them in Table 1.

Pre-transplant factors include older age, diabetes, and renal failure [15,18].

The patient’s nutritional status is also a risk factor: Kim et al. found that a low body mass index (BMI) was associated with an increased risk of bloodstream infections (BSIs) [19], while Park et al. demonstrated, through multivariate analysis, that a lower preoperative psoas muscle index was related to post-transplant bacteremia [20]. Hypoalbuminemia is also described in the literature as an additional risk factor for infections in liver transplant recipients [14].

Moreover, a high MELD score is associated with an increased risk of infection. Specifically, in the study by Chen et al., patients who developed post-transplant CRE infections were similar in terms of age, sex, BMI, and pre-transplant ICU stay compared to those without infection, but had significantly higher MELD scores [21]. Indeed, as a severity indicator, the MELD score is often associated with other conditions such as pre-transplant hospitalization and infections, ICU stay, and invasive procedures [9,15,19,22].

Heldman also demonstrated that pre-transplant infections are a risk factor for post-transplant infection, specifically post-transplant pneumonia and bloodstream infection [23].

Furthermore, colonization by MDROs is a major factor associated with the development of MDRO infections. This has been demonstrated for MRSA nasal colonization and VRE and CRE rectal colonization [24,25,26]. Frequent or prolonged hospitalization, exposure to invasive procedures or intravascular devices, and the use of broad-spectrum antimicrobials significantly increase the risk [13,27], especially the chronic use of quinolones for the prophylaxis of spontaneous bacterial peritonitis (SPB) [28]. Risk factors for MRSA nasal colonization include pre-transplant dialysis and a high MELD score [29]. Regarding CRE, Taimur’s study demonstrated a correlation between pre-transplant CRE colonization and/or infection and an increased risk of post-transplant infection, with higher risk seen when CRE was detected closer to the time of transplantation [25]. Evidence to the contrary, however, exists: Martin-Mateos et al. conducted a study with the aim of assessing the main risk factors associated with MDRO infection after LT, and, unexpectedly, the carrier status was not identified as an independent predictor of MDRO infection in multivariate analysis [30]. The risk factors specific to MDRO infections are summarized in Table 2.

Considering intraoperative factors associated with a higher risk of post-transplant infections, numerous studies in the literature have found a correlation between the duration of surgery, intraoperative blood loss, and the incidence of bacterial infections, especially surgical site infections and bloodstream infections [14,15]. In a multivariate analysis, Chen et al. documented how blood loss greater than 1500 mL during surgery was an independent risk factor for CRE infections [21], and Qian’s study observed that higher volumes of red blood cell (RBC) and plasma transfusions during surgery, a finding correlated with higher blood losses, were associated with post-transplant infections [18].

Furthermore, another factor that appears to play a role in the development of post-transplant infections is cold ischemia time (CIT). This is because it can cause biliary damage, leading to intra-abdominal and biliary complications and, in some cases, may necessitate surgical re-exploration, itself a risk factor for further infections [19,28].

Among post-transplant factors, some studies find a correlation between a prolonged stay in intensive care facilities and hospitals, post-transplant dialysis and mechanical ventilation, and the development of post-transplant infections [15]. A retrospective study by Chen et al. showed that patients with post-transplant vascular and biliary complications, who underwent mechanical ventilation for more than 72 h and renal replacement therapy for over 3 days, were more prone to CRE infection [21]. Moreover, the length of post-transplant hospital stay and post-transplant dialysis were associated with enterococcal bacteriemia [13].

In Wu’s study, multivariate analysis confirmed that 3 or more days of carbapenem therapy in the previous 15 days was an independent risk factor for post-transplant CRE infection; in addition, a significantly increased risk of Gram-negative infections was present in patients with a urinary catheter in place for more than 3 days [9].

Finally, EAD (early allograft dysfunction) and rejection also appear to be additional risk factors for post-transplant bloodstream infections: as the liver acts as a filter against intestinal bacteria, it easy to understand why EAD could be associated with an increased risk of early postoperative bacteremia [10,19,20].

## 5. Liver Transplant Recipients’ Prophylaxis

In guidelines from the American Society of Transplantation Infectious Diseases Community of Practice, the authors state that surgical antibiotic prophylaxis should be optimized for every single patient by employing a tailored regimen based on previous donor and recipient infections, MDRO colonization, and the organ transplant type [31].

There is no universal consensus about the use of surgical antibiotic prophylaxis or routine preoperative screening for MDRO colonization. Screening swabs are only performed in some centers, and the results often determine the choice of peri-transplant antibiotic prophylaxis. Several studies did not consider MDRO colonization at all for the choice of regimen as swabs were not performed [18,22,32,33]. A French retrospective study considered all the patients using an available pre-transplant rectal swab for ESBL-carrying *Enterobacteriaceae* (*n* = 749) (a demonstrated risk factor for post-transplant ESBL infection). The postoperative ESBL-related infection incidence after LT was significantly lower in patients who received active surgical prophylaxis against the colonized strains than in the others (29.8% versus 63.6%; *p* = 0.04) [34].

Perioperative prophylaxis is routinely administered, but there is no consensus in the literature about the optimal strategy and whether targeted or universal prophylaxis might be the most effective choice.

Two retrospective studies tried to compare different prophylactic regimens (i.e., carbapenems vs. cephalosporin/piperacillin-tazobactam) and both found no differences in the risk of SSIs or survival. The first was focused on patients with extremely advanced liver disease (MELD > 30) and who were therefore considered at higher risk of MDRO infection due to hospitalizations and procedures, but no screening for MDRO carriers was performed. The second compared regimens based on cephalosporins (ceftriaxone/cefazolin) vs. broad-spectrum prophylaxis (vancomycin plus aztreonam, piperacillin-tazobactam or carbapenems). Patients with pre-transplant MDROs more frequently received broad-spectrum coverage, but MDRO colonization was not associated with the development of SSIs [35,36].

Other studies, on the contrary, underline the role of MDRO colonization in post-transplant outcomes, highlighting the importance of MDRO screening and of targeted surgical prophylaxis [37]. For example, Sarwar et al. conducted a retrospective study on an exiguous number of patients (*n* = 27) and found that VRE-colonized recipients who received daptomycin did not develop VRE-related infections in the first 90 days post-LT [38]. Using a retrospective single-center study (*n* = 762), Freire et al. showed that SSIs caused by MDROs were associated with CRE colonization before LT and that the use of targeted surgical prophylaxis protected against SSIs caused by MDROs [39].

## 6. Expert Opinion

We provided an overview of clinically relevant evidence relating to early post-liver transplant bacterial infections, focusing specifically on MDRO etiology. This expert revision did not follow the structure of a formal systematic review but was instead based on a literature review intended to provide practical guidance. We performed no formal assessment of the strength or quality of the evidence.

We now use these insights to provide clinicians with recommendations relating to management and treatment in early liver transplant infections. These best practice recommendations are derived from a combination of available evidence and expert consensus.

(1) For patients showing clinical signs of bacterial infection, we recommend proceeding with a diagnostic work-up to identify the potential infection source and collect relevant microbiological samples in order to detect the causative pathogen. In liver transplant recipients, attention should be given to abdominal infections, surgical site infections, and bloodstream infections. We suggest the following measures:

- Close monitoring for signs indicative of peritonitis, ascites superinfections, cholangitis, and hepatic abscesses by employing radiological evaluation using abdominal ultrasound and/or CT scans.
- The surveillance of inflammatory signs at the surgical site.
- Monitoring invasive devices such as central venous lines, drainage sites, and bladder catheters.

Pathogen identification should include the collection of microbiological samples like blood cultures, urine cultures, and ascitic fluid cultures. Nasal and rectal swabs are also collected again, as are those obtained pre-transplant. Regarding donor-derived infections, an increasing amount of evidence suggests the importance of culturing graft preservation fluid [40].

**Figure 1:**
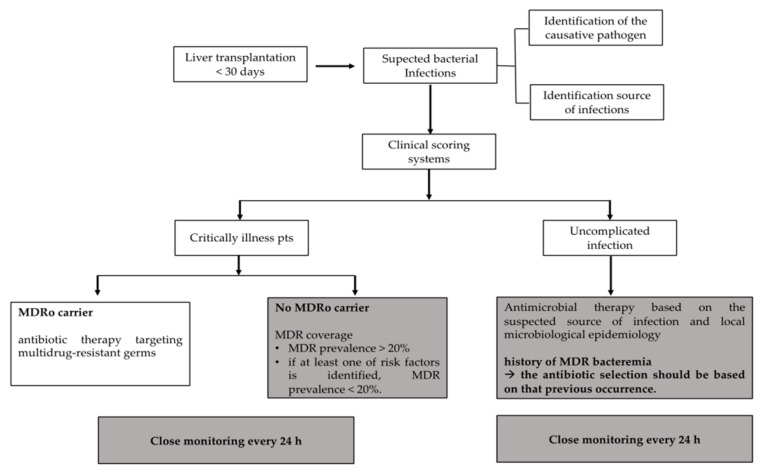
Algorithm for management and treatment considerations in early liver transplant infections.

(2) Several biochemical infection markers have been studied. You et al. conducted a meta-analysis and systematic review to assess the diagnostic accuracy of procalcitonin (PCT) as a biomarker for infection post-liver transplant [41]. The study concluded that while PCT is moderately accurate, its diagnostic performance remains suboptimal. Further research is necessary to explore new biomarkers that could be combined with PCT for a more rapid and precise diagnosis of postoperative infections. For example, Umman et al. found that both the red cell distribution width (RDW) and neutrophil-to-lymphocyte ratio (NLR) were associated with infection and were elevated before the detection of positive cultures [42].

(3) Active surveillance for the carriage of MDROs is generally considered to be a key moment in infection prevention and control. However, in the setting of liver transplantation, evidence regarding the role of screening in and its impact on the management of prophylaxis and treatment is scarce. Nevertheless, MDRO carrier status affects the antibiotic regimen choice, mainly in liver transplant patients with clinical manifestations that suggest the presence of severe life-threatening infections. The most controversial situations concern patients with severe infections but negative MDRO screening results, and those with uncomplicated infections and positive MDRO screening results. For the management of these “gray areas,” we propose an algorithm based on the risk of developing MDRO infection (Figure 1). In this, risk stratification is based on previously listed risk factors and screening carriage, as detailed below.

- For patients with severe clinical conditions but negative MDRO screening results, we suggest managing the infection on the basis of the local epidemiology. Antibiotics targeting MDROs should be chosen if the MDRO prevalence is greater than or equal to 20%, assuming none of the previously mentioned risk factors are present. If at least one of these risk factors is identified, MDRO coverage may be considered, even when the local prevalence is below 20%.
- For patients with positive MDRO screening results but uncomplicated infections (as assessed on the basis of clinical scoring systems like the NEWS—National Early Warning Score—or MEWS—Modified Early Warning Score [43]), overtreatment is a common risk, and so we recommend initiating antimicrobial therapy based on the suspected source of infection and local microbiological epidemiology, without necessarily considering the patient’s MDRO carrier status. In our opinion, a history of MDRO bacteremia constitutes an exception, as in this case antibiotic use should be based on that previous occurrence. Close monitoring of the patient is crucial in this instance, and it requires careful clinical and laboratory monitoring every 24 h. This ensures, on one hand, the proper implementation of antimicrobial stewardship practices (such as de-escalation therapy); on the other hand, it allows for ongoing reassessment and, if necessary, the broadening of the antimicrobial spectrum.

In confirmed MDR infections, options for treatment include β-lactam/β-lactamase inhibitor combinations (e.g., ceftazidime/avibactam), cefiderocol, tigecycline, fosfomycin, or aminoglycosides. Combination therapy may be considered in severe or resistant cases. Novel agents like monoclonal antibodies and bacteriophages are promising but require further validation in liver transplant populations. These therapeutic strategies should be implemented alongside antimicrobial stewardship programs to optimize outcomes and minimize resistance [11].

(4) The studies on prophylaxis are mostly single-center, retrospective, and not completely conclusive, especially given the highly variable MDRO prevalence seen among countries. Given the risk of increasing the prevalence of MDROs and spreading new strains in uncolonized areas, we suggest the implementation of local stewardship programs adapted to local epidemiological factors. Surgical prophylaxis must be optimized for every patient, identifying those at higher risk of developing MDRO infections, to reduce risks and enhance benefits. The optimal duration of peri-transplant prophylaxis should minimize exposure to unnecessary antibiotic burden, prevent therapy-related adverse events, and reduce the spread of MDROs. Thus, we suggest limiting antibiotic prophylaxis to 48 h after surgical procedure [30].

In conclusion, this review offers a practical synthesis of current evidence on early post-liver transplant bacterial infections, particularly those caused by MDROs. Work still needs to be performed. For example, a weighted score associated with post-LT MDRO infections could be a useful tool with which to guide the choice of prophylaxis. However, careful diagnostic workups, pathogen identification, and personalized antibiotic regimens already allow for effective management, ensuring both optimal patient outcomes and the responsible use of antimicrobial therapies. Ultimately, our approach aims to support clinicians in making informed treatment decisions while adhering to antimicrobial stewardship principles.

## Figures and Tables

**Table 1:** Risk factors for post-transplant bacterial infections.

Pre-Transplant Factors	Perioperative Factors	Post-Transplant Factors
Age (>45 yo)	Duration of surgery	ICU stay > 48 h
Diabetes	Intraoperative blood loss ≥1500 mL	Long hospital stay
Renal failure and dyalisis	Prolonged cold ischemia time	Indwelling devices
Malnutrition	Biliary complications	Early allograft dysfunction
High MELD score	Surgical re-exploration	Rejection
Pre-transplant infections		

**Table 2:** Risk factors for MDRO infections.

Risk Factors for MDRO Infections
MDRO colonization
High MELD score (>25)
Long ICU stay and hospitalization
Post-transplant renal failure requiring CRRT
Broad-spectrum antimicrobials use
Invasive procedures
Indwelling devices
Blood loss during surgery greater than 1.5 L
